# Crystal Structural and Functional Analysis of the Putative Dipeptidase from *Pyrococcus horikoshii* OT3

**DOI:** 10.1155/2009/434038

**Published:** 2009-06-28

**Authors:** Jeyaraman Jeyakanthan, Katsumi Takada, Masahide Sawano, Kyoko Ogasahara, Hisashi Mizutani, Naoki Kunishima, Shigeyuki Yokoyama, Katsuhide Yutani

**Affiliations:** ^1^Experimental Facility Division, SPring-8 Group, National Synchrotron Radiation Research Center, 101 Hsin-Ann Road, Hsinchu 30076, Hsinchu Science Park, Taiwan; ^2^Peptide Institute, Inc., Ina Mino-shi, Osaka 562-8686, Japan; ^3^RIKEN SPring-8 Center, Harima Institute, 1-1-1 Kouto, Sayo-cho, Sayo-gun, Hyogo 679-5148, Japan; ^4^RIKEN Systems and Structural Biology Center, Yokohama Institute, RIKEN, 1-7-22 Suehiro-cho, Tsurumi, Yokohama 230-0045, Japan; ^5^Graduate School of Science, The University of Tokyo, 7-3-1 Hongo, Bunkyo-ku, Tokyo 113-0033, Japan

## Abstract

The crystal structure of a putative dipeptidase (*Ph*dpd) from *Pyrococcus horikoshii* OT3 was solved using X-ray data at 2.4 Å resolution. The protein is folded into two distinct entities. The N-terminal domain consists of the general topology of the *α*/*β* fold, and the C-terminal domain consists of five long mixed strands, four helices, and two 3_10_ helices. The structure of *Ph*dpd is quite similar to reported structures of prolidases from *P. furiosus* (Zn-*Pf*prol) and *P. horikoshii* (Zn-*Ph*dpd), where Zn ions are observed in the active site resulting in an inactive form. However, *Ph*dpd did not contain metals in the crystal structure and showed prolidase activity in the absence of additional Co ions, whereas the specific activities increased by 5 times in the presence of a sufficient concentration (1.2 mM) of Co ions. The substrate specificities (X-Pro) of *Ph*dpd were broad compared with those of Zn-*Ph*dpd in the presence of Co ions, whose relative activities are 10% or less for substrates other than Met-Pro, which is the most favorable substrate. The binding constants of Zn-*Ph*dpd with three metals (Zn, Co, and Mn) were higher than those of *Ph*dpd and that with Zn was higher by greater than 2 orders, which were determined by DSC experiments. From the structural comparison of both forms and the above experimental results, it could be elucidated why the protein with Zn^2+^ ions is inactive.

## 1. Introduction

Prolidases (proline-specific dipeptidase) are peptidases with specificity for X-Pro dipeptides. X-Pro substrates contain N-terminal residues that are hydrophobic/uncharged (Ala-, Ile-, Leu-, Val-), basic (His-), aromatic (Phe-, Tyr-), or sulphur-containing (Met-) [1]. Prolidases only cleave dipeptides with proline at the C terminus (NH_2_-X-/-Pro-COOH). This modification or truncation process can develop either cotranslationally or posttranslationally after the action of an endoproteinase. Prolidase is widespread in nature and has been isolated from different mammalian tissues [2–4] as well as from bacteria, such as the species of *Lactobacillus * [1, 5] and *Xanthomonas * [6]. While the physiological role of prolidase in bacteria is unclear, a deficiency of this enzyme in humans results in abnormalities of the skin and other proline-rich collagenous tissues [7]. In contrast with other endopeptidases and exopeptidases, prolidase is thought to be involved in the terminal degradation of intracellular proteins, and may also function in the recycling of proline. Prolidase also has biotechnological applications; it has a potential use in the dairy industry as a cheese-ripening agent [8] because the degradation of proline-containing peptides in cheese reduces bitterness. Prolidases are also capable of detoxifying organophosphorus nerve agents such as sarin and soman [9].

 The crystal structure of prolidase has been solved only from *Pyrococcus furiosus * [10], where the main subunit is a “pita-bread” fold containing a metal active center like aminopeptidase P from *E. coli * [11] and methionine aminopeptidase from *P. furiosus * [12]. Two Zn atoms in the active site of the solved crystalline structure have been found [10], which are included as an impurity in the crystallization medium. However, the native prolidase from *P. furiosus * requires two Co ions per molecule in the active center for full catalytic activity. When Co ions are replaced by Zn ions, the protein does not show any enzymatic activity [13]. The structure of the prolidase containing Co ions with full activities remains to be solved.

 Recently, the structure of prolidase from *Pyrococcus horikoshii * OT3 (Project ID, PH1149), which has 80% sequence identities with that from *P. furiosus*, has been deposited in the Protein Data Bank (1WY2). This protein also has Zn ions in the active center as observed in the *P. furiosus * enzyme. Furthermore, when the structure of a protein annotated as a putative dipeptidase from *P. horikoshii* (Project ID, PH0974), having 36% sequence identities with PH1149, was solved, no metal ions were found in the active center. The protein showed substrate specific activities for a dipeptide of Met-Pro, which is a feature of X-Pro dipeptidase (prolidase).

In this paper, the structure of PH0974 (*Ph*dpd) is described in detail, and the difference in the structure of PH1149 with Zn ions (Zn-*Ph*dpd) will be discussed. In addition, the differences in both proteins in the binding feature of Co or Zn ions and in substrate-specific activities were examined in order to clarify the enzymatic function of this enzyme.

## 2. Materials and Methods

### 2.1. Expression and Purification

The gene was amplified by a polymerase chain reaction (PCR) using *P. horikoshii * OT3 genomic DNA as a template (Project ID: PH0974). Recombinant plasmid was constructed by the super-rare-cutter system (Hayashizaki et al., manuscript in preparation). *E. coli * BL21-CodonPlus (DE3)-RIL cells were transformed with the recombinant plasmid and grown at 37°C in LB medium containing 50 *μ*g mL^−1^ ampicillin for 20 hours. The cells were harvested by centrifugation at 6500 rpm for 5 minutes, suspended in 20 mM Tris-HCl, pH 8.0 (Buffer A) containing 0.5 M NaCl and 5 mM 2-mercaptethanol and disrupted by sonication. The cell lysate was heated at 90°C for 13 minutes. After heat treatment, denaturated proteins were removed by centrifugation (15,000 rpm, 30 minutes), and the supernatant solution was used as the crude extract for purification. The crude extract was desalted using a HiPrep 26/10 desalting column (Amersham-Biosciences) and applied onto a Super Q TOYOPEARL 650 M column (Tosoh) equilibrated with Buffer A. The protein was eluted with a linear gradient of 0–0.3 M NaCl in Buffer A. The protein was desalted with HiPrep 26/10 desalting column with Buffer A and subjected to a RESOURCE Q column (Amersham Biosciences) equilibrated with Buffer A. The protein was eluted with a linear gradient of 0–0.3 M NaCl in Buffer A. The buffer of the fractions containing the protein was exchanged using the HiPrep 26/10 desalting column to 10 mM sodium phosphate, pH 7.0 and applied onto a Bio-Scale CHT-20-I column (BIO-RAD) equilibrated with the same buffer. The protein was eluted with a linear gradient of 10–200 mM sodium phosphate, pH 7.0. The fractions containing protein were pooled, concentrated by ultrafiltration (VIVASPIN, 5 k cut) and loaded onto a HiLoad 16/60 Superdex 75 pg column (Amersham Biosciences) equilibrated with Buffer A containing 0.2 M NaCl. The purified protein showed a single band on SDS-PAGE. The concentration of the protein was estimated from the absorbance at 280 nm assuming *E*_1%_^1 cm^ = 10.14. This protein is abbreviated as *Ph*dpd.

Prolidase from *P. horikoshii * OT3 (Project ID: PH1149) was expressed and purified using similar methods. This protein also showed a single band on SDS-PAGE. The concentration of the protein was estimated from the absorbance at 280 nm assuming *E*_1%_^1 cm^ = 7.81. This protein is abbreviated as Zn-*Ph*dpd.

### 2.2. Crystallization

The protein concentration of *Ph*dpd subjected to crystallization was 20 mg mL^−1^ in 100 mM Tris buffer at pH 8.0 containing 0.2 M sodium chloride. Single crystals were grown using polyethylene glycol by the hanging drop vapor diffusion method. The reservoir contained 0.1 M buffer solution (Cacodylate—NaOH pH 6.5), 40%  (w/v) polyethylene glycol 400, and 0.02 M magnesium acetate. Each well was filled with 500 *μ*L of reservoir solution. Protein solution consisted of 1 *μ*L of a 20 mg mL^−1^ protein solution and 1 *μ*L of reservoir solution. The protein crystal used for data collection grew to the size of 0.1 × 0.1 × 0.1 mm after 8–10 days.

### 2.3. Data Collection and Processing

Diffraction data for *Ph*dpd were collected using a Rigaku R-AXIS V imaging-plate detector at the BL26B1 beamline, SPring-8, Japan. The crystals were flash-frozen in nitrogen-gas stream at 100 K during data collection. The oscillation angle used was 1.0° and the crystal-to-detector distance was set to 350 mm. Three data sets for the MAD (Multiwavelength Anomalous Dispersion) phasing were collected from a single selenomethionone-labelled crystal. Three wavelengths, corresponding to the maximum *f*′′ (peak), the minimum *f*′ (edge) and a reference wavelength (remote), were selected for the selenomethionine-labelled crystal, based on the fluorescence spectrum of the Se atom in the crystals. The wavelength was set to 1.0 Å for native crystals. The diffraction data were processed and scaled with the *HKL *2000 package [14].

### 2.4. Phase Determination and Refinement

**Figure 1:**
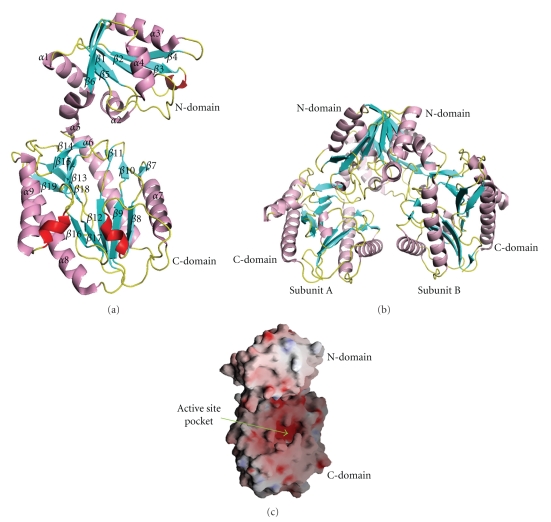
(a) Ribbon diagram of *Ph*dpd (monomer form) showing N and C-terminal domains. *α*-helix, 3_10_-helices, *β*-strands, and loops colored magenta, red, cyan, and yellow, respectively. The figure was made by the programs MOLSCRIPT and Raster3D [20, 21]. (b) Dimer form of *Ph*dpd. (c) Electrostatic potential surface of the *Ph*dpd and its active site pocket. The positively and the negatively charged surface regions are noted in blue and red, respectively.

The structure was solved by MAD method [15]. Se-atom positions were obtained with the program SOLVE and the initial electron density map was calculated by SOLVE and RESOLVE [16]. Phase calculation resulted in an overall figure of merit of 0.45 for data in the range of 20–2.6 Å resolutions. The program ARP/wARP [17] was used to automatically build a partial model of the dimeric enzyme based on the amino acid sequence to the MAD-phased electron density map at 2.6 Å and placed approximately 50% of the entire residues. Combined solvent flattening and histogram matching, as implemented in DM were used to improve the phases. Most of the secondary structure elements were interpretable in the improved map. Unambiguous parts and side chains could be added during the refinement, without noncrystallographic symmetry (NCS) restraints. The rest of the residues was built manually with QUANTA (Accelrys San Diego, Calif, USA). All crystallographic refinements were carried out using CNS 1.1 [18, 19]. Solvent molecules were gradually included into the structure at stereochemically preferred positions and with difference densities higher than 2.8*σ* (*F*_0_-*F*_*c*_) and 0.8*σ* (2*F*_0_-*F*_*c*_). A summary of the statistics for structure determination of *Ph*dpd is given in Table 1 and a ribbon diagram of the structure in Figure 1(a).

### 2.5. Analytical Ultracentrifugation

Sedimentation equilibrium experiments were carried out using a Beckmann Optima mode XL-A at 20°C with an An-60 Ti rotor at a speed of 13 K rpm. Prior to the measurements, the protein solutions were dialyzed overnight against the respective buffer at 4°C. The experiments at three different protein concentrations between 0.93 and 0.31 mg mL^−1^ were performed in Beckman 4-sector cells. The buffer used was 20 mM Tris, pH 8.0, including 100 mM NaCl. The partial specific volume of 0.751 cm^3^ g^−1^ used for *Ph*dpd was based on the amino acid compositions of the protein [22]. Analysis of the sedimentation equilibrium was performed using the program “XLAVEL” (Beckman, version 2.0).

### 2.6. Assay of Enzyme Activity

Proline dipeptidase activity was measured by a modification of the colorimetric ninhydrin method [24] using Met-Pro.HCl, Val-Pro.HCl, Gly-Pro.HCl, Ala-Pro.HCl, Phe-Pro.HCl, Glu-Pro.HCl, and Lys-Pro.HCl as substrates. Aminopeptidase and endopeptidase activities were measured by Met-MCA.TosOH (Tosylate form of L-methionine 4-methyl-coumaryl-7-amide) [25] and FRETS-25Xaa [26], respectively, as substrates. The FRETS-25Xaa is a fluorescence resonance energy transfer substrate (FRETS) library for determining endopeptidase specificity (Peptide Institute, Inc.). All assays were carried out at 100°C in 50 mM MOPS (3-[N-morpholino]propanesulfonic acid) buffer of pH 7.0, containing 1.2 mM CoCl_2_.

### 2.7. DSC Experiments

 DSC (differential scanning calorimetry) was carried out using a VP-capillary DSC platform (MicroCal, USA) at a scan rate of 100 deg h^−1^. The protein concentration in the measurements was fixed at 0.01 mM in 50 mM Tris, pH7.8. Metals (ZnCl_2_, CoCl_2_, and MnCl_2_) were dissolved in the buffer. The sample was filtered through a 0.22-*μ*m pore size membrane.

## 3. Results and Discussion

### 3.1. Quality of the Model

The structure of *Ph*dpd was determined by the MAD method at 2.3 Å resolution. The asymmetric unit contains two molecules, which are related by a two-fold noncrystallographic symmetry (NCS). The native structure was refined to an *R*-factor of 21%  (*R*_free_ = 26.5%) at 2.4 Å resolution. The root mean square deviations (rmsds) from ideal geometry for the bond lengths and bond angles were 0.008 Å and 1.4°, respectively. All residues are within allowed regions in the Ramachandran plot (93.8% in the most favored region). The stereochemistry of the refined structure was analyzed with the program PROCHECK [23]. The program LSQKAB [27] from CCP4 was used to calculate rms deviations for the superposition of molecules. A summary of the data collections, refined model and the relevant geometrical parameters is given in Table 1.

### 3.2. Description of the Structure

The final refined model consists of two complete polypeptide chains from Met1 to Leu356 and 310 ordered water molecules. Each of the monomer subunits has an N terminal domain (residues 1–120), an *α*-helical linker (residues 121–130) and a C terminal domain (residues 131–356). The overall topology of *Ph*dpd and a view of the C*α* backbone are shown in Figures 1(a) and 4(a). The N-terminal domain is composed of a central *β*-sheet with six *β*-strands (strand order: *β*4, *β*3, *β*2, *β*1, *β*5, and *β*6) and of five *α*-helices around the central *β*-sheet. The strands of *β*1 to *β*3 are in antiparallel relationship and the other strands are in parallel directions. The C-terminal domain is comprised of long mixed stranded *β*-sheets (*β*7–*β*19) with four *α*-helices (*α*6–*α*9), lying on the outside of the surface. The *α*-helices *α*6 and *α*8 run parallel to the nearby *β*-sheet, while helices *α*7 and *α*9 are in antiparallel relationship on the outside surface. This domain should be a catalytic domain, which is similar to the reported structures of the “pita-bread” fold [10, 12, 28–33].

The active center of *Ph*dpd can be assumed from the analogy of the structure of a “pita-bread” folded enzyme [34]. The putative active site pocket is located between two 3_10_ helixes (residues 191–195 and 281–284) (two red color helices in Figure 1(a)) and in a deep groove of the inner surface as shown in Figure 1(c). The active site is strongly curved by the central *β*-sheet of the C-terminal domain and stabilized by four helices (*α*6–*α*9) that cover the outside surface of the deep pocket. The N and C terminal domains are linked by an *α*5 helix (residues 121–130) spanning between *β*6 and *α*6.

### 3.3. Substrate Specificity

 Sequence identities of *Ph*dpd and Zn-*Ph*dpd from *P. horikoshii * are 36% (131/357), but those of Zn-*Ph*dpd and the prolidase (Zn-*Pf*prol) from *P. furiosus * are quite high, 80% (279/348). Therefore, Zn-*Ph*dpd has been assigned as a prolidase, although *Ph*dpd is a putative dipeptidase. We then examined the enzyme functions of *Ph*dpd and Zn-*Ph*dpd from *P. horikoshii. * Proline dipeptidase activities (X-Pro) of both proteins were the highest for the dipeptide Met-Pro among the substrates examined (Table 2). The specific activity of *Ph*dpd for the substrate Met-Pro was about 3 times that of Zn-*Ph*dpd. In the case of *Ph*dpd, the catalytic efficiencies for the peptide containing nonpolar amino acids were higher than those for the peptide containing polar amino acids such as Lys and Glu. The activity for Gly-Pro was the lowest. The substrate specificity of *Ph*dpd was broad compared with that of Zn-*Ph*dpd whose relative activities are 10% or less for substrates other than Met-Pro. The substrate specificities of Zn-*Ph*dpd are quite similar to the reported results for Zn-*Pf*prol as shown in Table 2. The effect of metal ions on the dipeptidase activities of *Ph*dpd and Zn-*Ph*dpd was examined using Met-Pro as a substrate. As shown in Table 3, the relative activity of *Ph*dpd in the presence of 1.2 mM MnCl_2_ was higher than that in 1.2 mM CoCl_2 _, but that of Zn-*Ph*dpd was about half. When the metal ions were not added, *Ph*dpd had 20% relative activity, but Zn-*Ph*dpd had none.

Kinetic parameters for Val-Pro of *Ph*dpd in the presence of 1.2 mM CoCl_2_ were determined to be 5.0 mM, 807 *μ*mol min^−1^ mg^−1^, 541 s^−1^, and 108 mM^−1^ s^−1^ for *K*_*m*_, *V*_max_, *k*_cat_, and *k*_cat_/*K*_*m*_, respectively. The value of *K*_*m*_ was similar to that reported for the prolidase from *P. furiosus * (Zn-*Pf*prol), but the other kinetic parameters of *Ph*dpd were several times greater [13].

The methionine aminopeptidase activity of *Ph*dpd was examined for Met-AMC. It was detectable, but the *V*_max_ value was less than 0.1% of that for the dipeptide Val-Pro. Furthermore, the endopeptidase activity was also examined with the substrate FRETS-25Xaa (Peptide Insititute, Inc.) which contains 475 combinations (25 × 19 = 475) of tripeptides except for cysteine. No endopeptidase activity was detected, even when 30 times the enzyme concentration was used compared with the assay for the dipeptide Val-Pro. These results indicate that *Ph*dpd can be called prolidase.

Because a cacodylate ion has been found the near active sites of Zn-*Ph*dpd, the dipeptidase activity using Met-Pro as a substrate was measured in the presence of cacodylate ion from 0.4 *μ*M to 40 mM. The results indicate that Zn-*Ph*dpd is not inhibited by cacodylate ion which was included in the crystalline buffer.

### 3.4. Changes in Denaturation Temperatures by the Addition of Metal Ions

**Figure 2:**
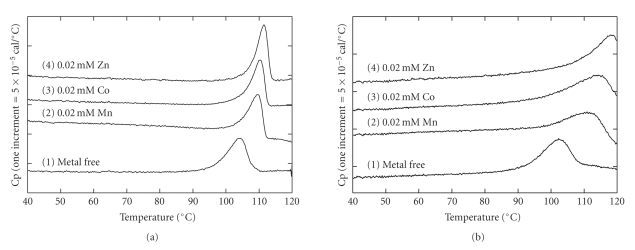
DSC curves of *Ph*dpd and Zn-*Ph*dpd in the presence of metal ions. (a) and (b) represent the DSC curves of *Ph*dpd and Zn-*Ph*dpd, respectively. All protein concentrations were 0.01 mM. DSC curves 1, 2, 3, and 4 were measured in the presence of 0 M metal, 0.02 mM Zn, 0.02 mM Co, and 0.02 mM Mn, respectively, in 50 mM Tris buffer at pH 7.8. The scan rate was 100 K h^−1^.

 The heat stability of a protein is enhanced by ligand binding. Using DSC, the binding constant between a protein and a ligand can be estimated from the shift in the denaturation temperature for thermal unfolding of a protein in the presence of a ligand relative to the denaturation temperature in the absence of the ligand [35]. Figure 2(a) shows the DSC curves of *Ph*dpd in the presence of metals whose concentrations are twice that of the protein where two metal-binding sites in the protein are saturated. The peak temperature of the DSC curve in the absence of the metal was 104.4°C and was lower than those in the presence of metals (Table 4), indicating that these metals can tightly bind to *Ph*dpd. The difference in peak temperatures between the proteins in the absence and presence of metals indicates that Zn ion most strongly binds to the protein, followed by Co and Mn ions (Figure 2(a), Table 4). In the case of Zn-*Ph*dpd, as shown in Figure 2(b), the order of binding strength for three kinds of metals to the protein was similar to that of *Ph*dpd, but the strength seemed to be considerably higher than that of *Ph*dpd: the differences in peak temperatures between the proteins in the absence and presence of 0.02 mM Co were 5.9 and 13.2°C for *Ph*dpd and Zn-*Ph*dpd, respectively (Table 4). The DSC curves of *Ph*dpd and Zn-*Ph*dpd, which were dialyzed in 50 mM Tris buffer at pH 7.8 including 1 mM EDTA overnight, were similar to those of samples without the metal ion. This suggests that original proteins hardly bound metal ions such as Zn, Co, and Mn.

**Figure 3:**
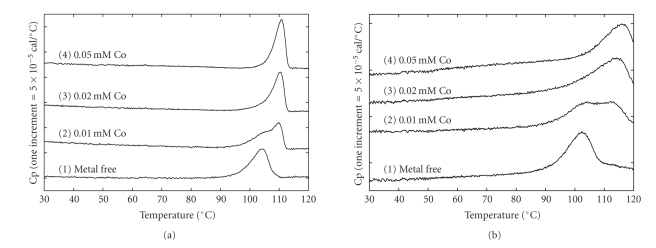
DSC curves of *Ph*dpd and Zn-*Ph*dpd in various concentrations of Co ion. (a) and (b) represent the DSC curves of *Ph*dpd and Zn-*Ph*dpd, respectively. All protein concentrations were 0.01 mM. DSC curves 1, 2, 3, and 4 were measured in the presence of 0, 0.01, 0.02, and 0.05 mM Co, respectively, in 50 mM Tris buffer at pH 7.8. The scan rate was 100 Kh^−1^.

 Reheating the DSC curves of *Ph*dpd and Zn-*Ph*dpd did not show any excess heat capacities, indicating that heat denaturation of both proteins is irreversible. Therefore, it might be difficult to strictly analyze the binding constants from the shifts in peak temperature due to ligand binding. After an error margin had been agreed upon, we calculated the binding constants using estimated DSC parameters and changes in denaturation temperatures because these are considerably more reliable. In the presence of 0.02 mM Zn ions, the binding constants of *Ph*dpd and Zn-*Ph*dpd were 1.2 × 10^7^ and 1.6 × 10^9^ M^−1^, respectively. The results suggest that the binding constants of Zn-*Ph*dpd with Zn ions were roughly higher than 2 orders compared to those of *Ph*dpd (Figures 3(a) and 3(b)).

On the other hand, methionine aminopeptidase from *E. coli, * which has a “pita-bread” fold with two active metal sites, has been reported to be maximally stimulated with the addition of one equivalent of Co^2+^ or Fe^2+^, and the first metal ion binds with a binding constant of 3–5 × 10^6^ M^−1^, while the second one binds at 0.4 × 10^3^ M^−1^ based on the changes in the absorption spectra during titration [36].

### 3.5. Comparison of Structures Near Active Sites of Phdpd with Those of Zn-Phdpd

The crystal structure and amino acid sequence of the prolidase from * P. horikoshii * (Zn-*Ph*dpd) (PDB ID: 1WY2) are quite similar to those of that from * P. furiosus * (Zn-*Pf*prol) (PDB ID: 1PV9). Both proteins have two Zn ions in the active sites resulting in the absence of function. On the other hand, *Ph*dpd with the sequence identity of 38% to Zn-*Pf*prol and Zn-*Ph*dpd showed prolidase activity in the absence of additional Co ions (Table 3). Therefore, to elucidate why the proteins containing Zn ions do not have prolidase activity without the addition of Co ions, the structures of *Ph*dpd, Zn-*Ph*dpd, and Zn-*Pf*prol were compared.

**Figure 4:**
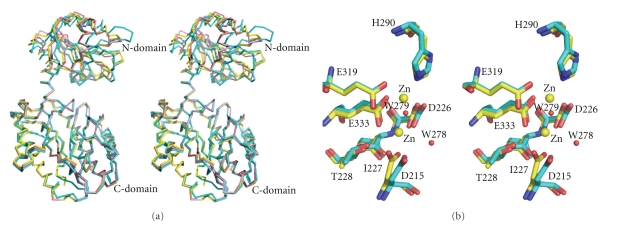
(a) Stereoview of the superposition of *Ph*dpd (Cyan) with Zn-*Ph*dpd (Yellow) and Zn-*Pf*prol (Pink) structures. (b) Stereoview of the conserved active site residues superimposed between the *Ph*dpd (cyan) and Zn-*Ph*dpd (yellow). Two active site metal atoms of Zn-*Ph*dpd and two water molecules (W278 and W279) of *Ph*dpd are shown in yellow and red color spheres, respectively. The side chains of the conserved active site residues are labeled according to *Ph*dpd sequence numbering.

**Figure 5:**
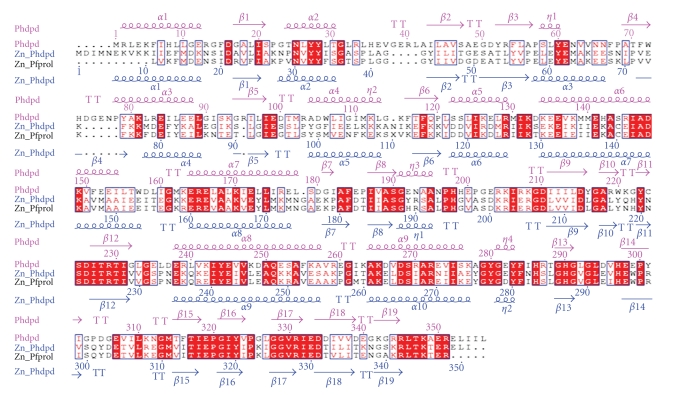
Structure-based sequence alignment of dipeptidase (PDB ID: 2HOW from *P. horikoshii OT3 * (*Ph*dpd) with prolidase from *P. furiosus * (Zn-*Pf*prol) and dipeptidase (PDB ID: 1WY2) from *P. horikoshii OT3 * (Zn-*Ph*dpd)*. * Above and below the alignment are shown the secondary structure elements. Alignment was performed using CLUSTAL W [37], and the figure was produced using ESPript [38].

**Figure 6:**
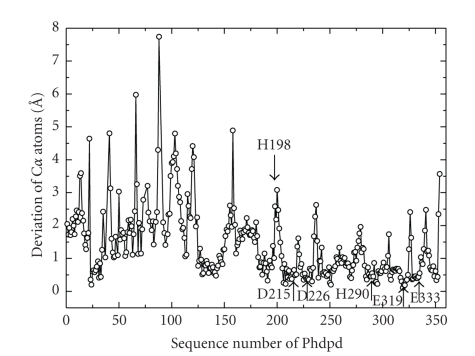
Deviations of C*α* atoms after the C*α* superposition between *Ph*dpd and Zn-*Ph*dpd. Downward and upward arrows represent the positions of H198, D215, D226, H290, E319, and E333 which are important residues in the active site.

Figure 4(a) shows a stereoview of the superposition of *Ph*dpd with Zn-*Ph*dpd and Zn-*Pf*prol structures. Furthermore, structure-based sequence alignment of the three proteins and rms deviation of C*α* atoms between *Ph*dpd and Zn-*Ph*dpd are shown in Figures 5 and 6, respectively. Comparison of *Ph*dpd with Zn-*Ph*dpd and Zn-*Pf*prol reveals major differences in folding, size, insertions and positioning of secondary structure elements in the N-terminal domain (Figure 5). In particular, the 3_10_ helix *η*1 is replaced by an *α*3 helix (residues: 57 to 67) in both Zn-*Ph*dpd and Zn-*Pf*prol (Figure 5). The rms deviation from C*α* superposition of the whole, N- and C-terminal domains was calculated separately as follows: 1.4, 2.3, and 1.0 Å for the superposition between Zn-*Pf*prol and *Ph*dpd, respectively; and 1.5, 2.0, and 1.1 Å for that between Zn-*Ph*dpd and *Ph*dpd, respectively (Figure 4(a))*. * Rms deviation values of five conserved residues (Asp215, Asp226, His290, Glu319, and Glu333) and the neighboring two residues (Ile227 and Thr228) belong to the lowest group of rms deviation values as shown in Figure 6, suggesting that these seven residues are considerably important in the active center. Figure 4(b) shows a stereoview of the conserved active site residues superimposed between the *Ph*dpd and Zn-*Ph*dpd. A cacodylate ion was found close to the active site of Zn-*Ph*dpd, but not in that of *Ph*dpd although both proteins were crystallized in the buffer containing cacodylate ions. A structural comparison of *Ph*dpd with Zn-*Ph*dpd reveals that the metal-coordination sphere and stereochemical organization of the active site are slightly altered due to Zn^2+^ binding as shown in Figure 4(b). Five water molecules (wat133, wat268, wat278, wat279 and wat290) are located around the active site pocket in *Ph*dpd, all of which form a hydrogen-bonding network. The wat279 molecule nearly occupies the place of one of zinc ions in Zn-*Ph*dpd. The water molecule w279 also creates similar coordination distances with conserved active site residues of *Ph*dpd and Zn-*Ph*dpd. The coordination distances of Asp226, Glu333, Ile227, and Thr228 of *Ph*dpd are slightly different from those of Zn-*Ph*dpd (Table 5). These observations might be correlated with the differences in binding with Zn and Co ions in the active site pocket.

**Figure 7:**
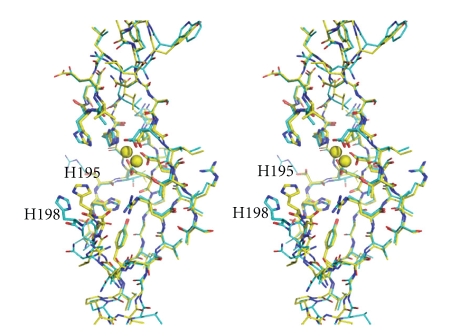
Stereoview of selected active site pocket residues of *Ph*dpd (cyan) superimposed on Zn-*Ph*dpd (yellow). Active site pocket residues are shown by the ball and stick model and the metal ions are shown as spheres. His195 of Zn-*Ph*dpd corresponds to His198 of *Ph*dpd.

It has been proposed that methionine aminopeptidase and aminopeptidase P, which involve the “pita-bread” fold that contains Co or Mn ions in the active site, have a common reaction mechanism [34]. The important active site residues interacting with substrates are conserved in the three proteins described above. One of them, His198 of *Ph*dpd corresponds to His79 of methioneine aminopeptidase from *E. coli*, which interacts with the nitrogen atoms of the scissile peptide bonds. Mutation of this residue of methionine aminopeptidase and aminopeptidase P has been reported to lead to variants with negligible activities [39, 40]. As shown in Figures 6 and 7, the position of His198 of *Ph*dpd is remarkably different from that of the corresponding His195 of Zn-*Ph*dpd: the rms deviation value of C*α* atoms of both proteins was 2.59 Å as represented by an arrow to His198 in Figure 7. Relocation of this His residue tends to decrease the volume of the active site pocket. These results indicate that the absence of activity of Zn-*Ph*dpd containing Zn ions might be caused by changes in coordination geometry of the metal ions and/or relocation of an important active side residue.

### 3.6. Structure of the Dimer and Dimer Interface

Analytical centrifugation results showed that *Ph*dpd exists as a dimer in solution, and the association constant of monomer/dimer is 1.6 × 10^6^ M. As shown in a ribbon diagram of Figure 1(b), the dimer form of *Ph*dpd is an assembly of two monomers, related by a noncrystallographic 2-fold axis. The asymmetric unit also contains two molecules in the crystal as well as a dimer in solution. The dimeric enzyme has an overall globular shape of approximately 55 Å × 80 Å × 61 Å with a depression at its center. The accessible surface area of the monomer subunit is 16037 and 15955 Å^2^ for the respective subunits A and B. The area buried due to a dimer formation was 2310 Å^2^ and 7.2% of the total surface area. The buried surface area of *Ph*dpd was remarkably smaller than those of Zn-*Ph*dpd and Zn-*Pf*prol; especially, the difference in buried area of nonpolar atoms was remarkably great between them as shown in Table 6. When the buried area is divided into nonpolar (C/S) and polar (N/O) atoms, the hydrophobic interaction of dimer formation (Δ*G*_HP_) can be estimated using the following equation:

(1) $$\Delta G_{\text{HP}}=\alpha \Delta {\text{ASA}}_{\text{nonpolar}}+\beta \Delta {\text{ASA}}_{\text{polar}},$$

where ΔASA_nonpolar_ and ΔASA_polar_ represent the difference in ASA (accessible surface area) due to dimer formation of the nonpolar and polar atoms of all residues, respectively. Parameters *α* and *β* have been determined to be 0.154 and −0.026 kJ mol^−1^ Å^−2^, respectively, using the stability/structure database of mutant human lysozymes [41]. The great differences in ΔASA values of nonpolar atoms between *Ph*dpd and Zn-*Ph*dpd (or Zn-*Pf*prol) resultantly indicate that hydrophobic interaction (Δ*G*_HP_) due to dimer formation of Zn-*Ph*dpd and Zn-*Pf*prol is remarkably higher than that of *Ph*dpd (Table 6).

### 3.7. Prolidases from P. horikoshii

One of two prolidases from *P. horikoshii, * Zn-*Ph*dpd, has considerably high sequence identities with the prolidase (Zn-*Pf*prol) from *P. furiosus*, but the corresponding gene to *Ph*dpd is not found in the genome of *P. furiosus*. In the process of BLAST searches [42], we found that a hypothetical protein (PH1902) from *P. horikoshii * can be annotated as X-pro dipeptidase from its 91% sequence identity with the X-pro dipeptidase from *Pyrococcus absysi*, which has identities of 26% and 28% with *Ph*dpd and *Zn*-Phdpd, respectively. The sequence of PH1902 has 29% identity with Zn-*Pf*prol but has higher identity with the other two prolidases from *P. furiosus * (76 and 58%). Furthermore, PepQ-3 X-pro aminopeptidase and PepQ-2 cobalt-dependent proline dipeptidase from *Pyrococcus abyssi * have high sequence identities with *Ph*dpd (76%) and Zn-*Ph*dpd (84%), respectively, and two different X-pro aminopeptidases from *Thermococcus kodakarensis * have 71 and 63% identities with *Ph*dpd and Zn-*Ph*dpd, respectively. There are several prolidases in each genome.

Although the physiological role of prolidases in a cell remains to be solved, the substrate specificity of *Ph*dpd is broader and its function is more effective than that of Zn-*Ph*dpd. The active site structures of both proteins are changed to active or inactive forms depending on the binding metals. When both active and inactive structures of each prolidase are solved in the future, the role of metal ions on the function of metalloaminopeptidases could be more clearly elucidated. 

## 4. Conclusions

The enzyme assay of Project ID PH0974 (*Ph*dpd) of *P. horikoshii * indicated that *Ph*dpd has the function of X-pro dipeptidase (prolidase). The crystal structure of *Ph*dpd was solved at 2.4 Å resolution, and there are no metal ions in the active site. Furthermore, DSC experiments suggest that there are big differences in binding constants with Zn between *Ph*dpd and Zn-*Ph*dpd. In order to elucidate why the proteins containing Zn ions do not have the prolidase activity without the addition of Co ions, the three structures of *Ph*dpd, Zn-*Ph*dpd, and Zn-*Pf*prol were compared. The conclusions were (1) the coordination geometry in the active site of *Ph*dpd was slightly different from that of Zn-*Ph*dpd and (2) the important His residue of Zn-*Ph*dpd, which seems to interact with the nitrogen atoms of the scissile peptide bonds, considerably moved resulting in decreasing the volume of the active site pocket due to Zn binding.

## Figures and Tables

**Table 1:** Data collection and refinement statistics of *Ph*dpd.

	Native	MAD (Se)
Data collection and phasing		
Wavelength (Å)	1.0000	0.97929, 0.97950, 1.0000
Temperature (K)	100	100
Space group	*P*2_1_2_1_2_1_	*P*2_1_2_1_2_1_
Unit cell dimensions		
a (Å)	57.9	57.8
b (Å)	88.8	88.8
c (Å)	147.6	147.1
*V*_*m*_ (Å^3^ Da^−1^)	2.4	2.3
No. of molecules in asu	2	2
Resolution (Å)	2.4 (2.5-2.4)	2.3 (2.38–2.3)
No. of observations	182 560	192 019, 190 610, 177 795
No. of unique reflections	29 349 (2954)	34 548, 34 494, 33 486
Completeness (%)	95.9 (98.6)	99.3 (99.9), 99.2 (99.9), 96.5 (98.4)
*R*_merge_ (%)^(a)^	11.2 (37)	7.0 (34.4), 5.7 (30.9), 5.2 (28.7)
FOM(Solve/Resolve)^(b)^	—	0.45/0.57

Refinement		
Resolution range (Å)	20.0–2.4	
*R*_work_^(c)^	21.0	
*R*_free_^(d)^	26.5	
No. of protein atoms	5664	
No. of solvent atoms	310	
Ramachandran plot^(e)^ (%) in most favored	93.8	
Allowed	5.7	
Generously	0.5	
PDB code	2HOW	

^(a)^ *R*_merge_ = ∑|*I*_*i*_ − 〈*I*〉|/∑*I*_*i*_, where *I* is the observed intensity and 〈*I*〉 is the average intensity from observations of symmetry related reflections, respectively.

^(b)^FOM, figure of merit.

^(c)^ *R*_work_ = ∑|*F*_obs_ − *F*_calc_|/∑|*F*_obs_|, where *F*_obs_ and *F*_calc_ are the observed and calculated structure factors, respectively.

^(d)^ *R*_free_ is the *R* factor for a subset of 5% of the reflections that were omitted from refinement.

^(e)^As calculated by PROCHECK [23].

The values within the parentheses refer to the last shell.

**Table 2:** Substrate specificity of three prolidases from *P. horikoshii * and * P. furiosus.*

Substrates	Relative activity (%)		
	*Ph*dpd	Zn-*Ph*dpd	Zn-*Pf*prol^(f)^
Met-Pro.HCl^(a)^	100	100	100
Val-Pro.HCl^(a)^	53	4	10
Ala-Pro.HCl^(a)^	35	7	17
Glu-Pro.HCl^(a)^	28	5	(e)
Phe-Pro.HCl^(a)^	24	10	24
Lys-Pro.HCl^(a)^	17	0	10
Gly-Pro.HCl^(a)^	2	(e)	1
Met-MCA.TosOH^(b)^	<0.1	(e)	(e)
FRETS-25Xaa^(c)^	0^(d)^	(e)	(e)

^(a)^The specific activity in the presence of 1.2 mM CoCl_2_ is expressed as a percentage of the activity compared to that obtained with Met-Pro. The average values of three experiments are listed.

^(b)^ *V*_max_ was compared to that obtained with Val-Pro.

^(c)^FRETS is a fluorescence resonance energy transfer substrate library for determining endopeptidase specificity (Peptide Institute, Inc.).

^(d)^The endopeptidase activity was not detectable.

^(d)^ Not examined.

^(e)^ Reported results [13].

**Table 3:** Effect of metal ions on dipeptidase (Met-Pro) activity of *Ph*dpd and Zn-*Ph*dpd.

Metal (1.2 mM)	Relative activity (%)	
	*Ph*dpd	Zn-*Ph*dpd
CoCl_2_	100	100
MnCl_2_	155	53
ZnCl_2_	1	1
no metal	20	1

Zn-*Ph*dpd was not inhibited in the presence of cacodylate ion from 0.4 *μ*M to 40 mM concentrations.

Specific activity of *Ph*dpd in the presence of 1.2 mM Co ions was 3 times that of Zn-*Ph*dpd.

**Table 4:** Changes in denaturation temperatures for *Pf*dpd and Zn-*Pf*dpd in the presence of metal ions at pH 7.8.

(mM)	*Ph*dpd		Zn-*Ph*dpd	
	*T*_*m*_ (°C)	Δ*T*_*m*_	*T*_*m*_ (°C)	Δ*T*_*m*_
Zn				
0.05			118.4	16.9
0.02	111.6	7.2	118.4	16.9
0.01	110.9	6.5	118.4	16.9

Co				
0.05	110.7	6.3	116.5	15.0
0.02	110.3	5.9	114.7	13.2
0.01	109.9	5.5	112.9	11.4

Mn				
0.05	110.1	5.7	114.5	13.0
0.02	109.7	5.3	112.2	10.7
0.01	109	4.6	109.8	8.3

The denaturation temperatures (*T*_*m*_) were obtained from the peak temperatures of DSC curves. The *T*_*m*_ values of *Pf*dpd and Zn-*Pf*dpd in the absence of metals were 104.4 and 101.5°C, respectively. All protein concentrations were 0.01 mM.

**Table 5:** Comparison of the active site coordination distances between *Ph*dpd and Zn-*Ph*dpd. The distances of Zn1 and Zn2 in *Ph*dpd were calculated after the superposition between atoms of the active site residues of *Ph*dpd and Zn-*Ph*dpd.

Distances in *Ph*dpd (Å)					Distances in Zn-*Ph*dpd (Å)		
Residues	Zn1	Zn2	W278	W279	Residues	Zn1	Zn2

Asp215 OD1	2.3		3.9		Asp212 OD1	2.0	
Asp215 OD2	3.4		2.8		Asp212 OD2	2.6	
Asp226 OD1		2.3		2.9	Asp223 OD1		2.0
Asp226 OD2	2.0			3.4	Asp223 OD2	2.1	
His290 NE2		2.5		3.5	His287 NE2		2.1
Glu319 OE1		3.1		2.8	Glu316 OE1		3.2
Glu319 OE2		2.3		3.1	Glu316 OE2		2.2
Glu333 OE1	2.2			3.6	Glu330 OE1	2.0	
Glu333 OE2		1.9		3.2	Glu330 OE2		2.1
Ile227 O	3.5				Ile224 O	4.2	
Thr228 OG1	3.0				Thr225 OG1	3.5	
W278	2.6	1.8		3.6	Zn1		3.2
W279	4.2	5.1					

**Table 6:** Comparison of hydrophobic interaction due to dimer formation of A and B subunits for *Ph*dpd, Zn-*Ph*dpd, and Zn-*Pf*prol.

	*Ph*dpd	Zn-*Ph*dpd	Zn-*Pf*prol	Δ(=*Ph*dpd-Zn-*Ph*dpd)	Δ(=*Ph*dpd-Zn-*Pf*prol)
ΔASA_np_ = (monomer* − dimer)	1396	2341	2263	−945	−867
ΔASA_p_ = (monomer* − dimer)	914	1067	1070	−153	−156
Total buried area (Å^2^)	2310	3408	3333		
Δ*G*_HP_ (kJ/mol)	191	333	320	−142	−129

*Monomer means the summation of ASA of A- and B-subunits.

ASA_np_ and ASA_p_ represent accessible surface area of nonpolar (C and S) atoms and polar (N and O) atoms, respectively.

The ASA values were calculated using software [20]. The unit of the ASA value is Å^2^ . Δ*G*_HP_ was calculated using (1).

## Abbreviations

*Ph*dpd: X-Pro dipeptidase (prolidase) of Project ID PH0974 from *P. horikoshii*
Zn-*Ph*dpd: X-Pro dipeptidase (prolidase) of Project ID PH1149 from *P. horikoshii*
Zn-*Pf*prol: Prolidase from *P. furiosus*
DSC: Differential scanning calorimetry.
